# The anti-inflammatory effects of dexamethasone implants in eyes with heavy silicone oil: a prospective study on vitrectomy for diabetic retinopathy

**DOI:** 10.3389/fendo.2025.1673884

**Published:** 2025-10-17

**Authors:** Xingguo Zhang, Junliang Dong, Haomin Lu, Minghao Sun, Shuning Xu, Yuege Zhang, Yi Peng, Dawei Sun, Bo Jiang

**Affiliations:** ^1^ Department of Ophthalmology, The Second Affiliated Hospital of Harbin Medical University, Harbin, China; ^2^ Department of Ophthalmology, Harbin Second Hospital, Harbin, China

**Keywords:** diabetic retinopathy1, diabetic tractional retinal detachment2, dexamethasone implants 3, pars plana vitrectomy4, heavy silicone oi5

## Abstract

**Purpose:**

This study aims to assess the effects of heavy silicone oil (HSO) tamponade in patients with diabetic tractional retinal detachment (TRD) treated with pars plana vitrectomy (PPV), and to evaluate the role of extended-release hormone implantation in controlling inflammation and mitigating postoperative complications.

**Methods:**

A prospective, randomized, controlled design was used in this study. This study included 96 eyes from 96 patients diagnosed with diabetic TRD, all of whom were treated at our institution between March 2024 and March 2025. Group 1 consisted of 54 eyes from 54 patients (PPV + HSO + dexamethasone implant), while Group 2 included 42 eyes from 42 patients (PPV + HSO). The primary outcomes included best-corrected visual acuity (BCVA), intraocular pressure (IOP), retinal reattachment rate, anterior chamber (AC) fibrin exudation, anterior proliferative vitreoretinopathy (PVR), and posterior iris synechiae during the 3-month postoperative follow-up period.

**Results:**

Eighty eyes from 80 patients were ultimately included in the study, with 40 eyes assigned to each group. Patient characteristics, including age, sex, preoperative HbA1c levels and preoperative blood pressure,DR severity were similar (*P >*0.05). Both groups demonstrated improvements in BCVA postoperatively compared to baseline (*P <*0.05,Bonferroni adjusted). Group 1 demonstrated a improvement in BCVA compared to Group 2 at 1 month and 3 months postoperatively,but *P* >0.05 after adjusted. At 3 months postoperatively, IOP did not differ significantly between the groups (*P* > 0.05). At 1 week postoperatively, AC fibrin exudation was decreased in Group 1(*P* = 0.007, *P* = 0.07 Bonferroni adjusted). At 3 months postoperatively,compared with Group 2, Group 1 had significantly lower postoperative anterior PVR (*P* = 0.010 Bonferroni adjusted), Group 1 had lower rates of retinal reattachment and posterior iris synechiae (*P* = 0.032 and *P* = 0.008, all *P*>0.05 Bonferroni adjusted).

**Conclusion:**

To our knowledge, this first study of dexamethasone implants with HSO in PDR suggests intraoperative DEX may be a safe, potentially beneficial adjunct for diabetic TRD undergoing phacoemulsification plus vitrectomy with HSO, reducing inflammation and number of anti-VEGF injections over 3 months, and offering useful directions for future research in this preliminary prospective evaluation overall.

## 1 Introduction

Diabetes is one of the primary contributors to vision impairment in adults within the working-age group (1). The progression of diabetes can lead to a wide range of irreversible complications; for example, one-third of diabetic patients suffer from diabetic retinopathy (DR) (2). VEGF plays a crucial role in this process. Neovascularization can occur when VEGF levels are elevated, which is a sign of proliferative diabetic retinopathy (PDR). PDR is among the most serious complications of diabetes, often resulting in permanent vision loss (3, 4). Additionally, numerous studies have shown that increased levels of inflammatory factors promote angiogenesis and neuronal degeneration, leading to retinal damage; therefore, inflammatory factors are crucial in the early development of DR (5). As the DR progresses, patients may develop diabetic macular oedema (DME), vitreous hemorrhage (VH), and TRD and even progress to neovascular glaucoma. Pars plana vitrectomy (PPV) is an important method for treating complications of PDR and refractory macular oedema (6, 7). During surgery, different types of tamponade (e.g., gas, silicone oil) are chosen based on the presence of retinal tears and tractional retinal attachment. However, according to the literature, silicone oil can induce an intraocular inflammatory response, which tends to intensify over time (8). Increased intraocular inflammatory factor levels are strongly associated with the onset and progression of PVR (9, 10). Although the fibrovascular membranes are removed and the detached retina is reattached during PPV, postoperative complications such as haemorrhage, retinal detachment (RD), PVR, and macular oedema may still occur. Anti-VEGF therapy may still be required in some patients after PPV to alleviate DME (11), however, there is currently no effective method to prevent the onset of retinal proliferation.

The application of intravitreal extended-release hormones to treat diabetic macular oedema primarily offers anti-neovascularization, anti-inflammatory, and anti-osmotic effects. Compared with other anti-VEGF drugs, intravitreal extended-release hormones can provide sustained release for up to 6 months after intravitreal administration (12, 13). Clinical studies have shown that the implantation of extended-release hormones can significantly reduce the recurrence rate of RD, minimize the severity of PVR, and decrease the need for anti-VEGF treatments in DME patients during PPV (14–16). However, there are no clinical studies examining the combination of HSO tamponade with extended-release hormone implantation during PPV in patients with PDR. The primary objective of this study was to assess the effects of HSO tamponade in patients with diabetic TRD undergoing PPV and to evaluate the role of extended-release hormone implantation in controlling inflammation and mitigating postoperative complications. This study will observe these contents.Although dexamethasone implants are widely used in patients with diabetic retinopathy, this study will provide a new treatment option for diabetic retinopathy patients who require heavy silicone oil filling during surgery.

## 2 Materials and methods

The study was conducted as a randomized, prospective, controlled design at the Second Affiliated Hospital of Harbin Medical University, with ethical compliance ensured through Helsinki Declaration adherence.The Institutional Review Board of Harbin Medical University granted ethical approval for this study (Study No. YJSKY2024-334). All enrolled subjects voluntarily provided signed informed consent documents in compliance with ethical requirements. This study included 96 eyes from 96 patients diagnosed with diabetic TRD, all of whom were treated at our institution between March 2024 and March 2025. A computer-based randomization system (Research Randomizer) was used to randomly assign numbers to the subjects. We placed the generated randomization sequence into sequentially numbered, sealed, opaque envelopes. Subsequently, personnel not involved in subject screening opened these envelopes in sequential order to implement the group assignment. These patients received a 25G three-port PPV under retrobulbar anaesthesia. Group 1 received PPV with HSO and dexamethasone implantation, whereas Group 2 underwent PPV with HSO only.

### 2.1 Patient eligibility

Inclusion criteria comprised the following: (1) diagnosis of type 2 diabetes mellitus, (2) participants aged 40 years or older, (3) TRD due to PDR requiring HSO tamponade, with concurrent cataracts or intraocular lenses (IOLs), and (4) at least 3 months of postoperative follow-up.

Exclusion criteria comprised: (1) incomplete clinical follow-up (<3 months); (2) TRD not caused by PDR; (3) patients presenting with exudative retinal detachment or rhegmatogenous retinal detachment; (4) the presence of glaucoma and an IOP more than 21 mmHg; (5) patients with other retinal pathologies that could affect macular structure and vision; (6) patients who had undergone vitreoretinal surgery; (7) cases exhibiting iris neovascularization; (8) patients with uncontrolled diabetes (glycosylated haemoglobin [HbA1c] >12%); and (9) patients with bleeding disorders, active ocular infections, a history of recent myocardial infarction, or pregnancy.

### 2.2 Sample size estimation

The primary endpoint of this study is the incidence of PVR (binary outcome). We will use a two-sample comparison of proportions (superiority, two-sided design). The significance level is set at α = 0.05 (two-sided) with Power = 0.80, and the allocation ratio is 1:1. Based on prior studies of dexamethasone in PDR patients undergoing silicone oil tamponade, we assume a PVR incidence of 28.6% in the control group (no dexamethasone implant) and 0% in the treatment group, with an anticipated loss to follow-up rate of 10%. The required sample size was calculated to be 50 participants.

### 2.3 Examinations

A detailed patient history, including sex, age, blood pressure, and blood glucose levels, was collected preoperatively. The complete ophthalmologic examinations were performed on all participants, which included measurement of BCVA (logarithm of the minimum angle of resolution [logMAR]), dilated fundus examination using a slit-lamp biomicroscope, IOP measurement via a noncontact tonometer, and measurement of central macular thickness (CMT) in the central 1-mm-diameter region using spectral-domain optical coherence tomography (OCT). Additionally, B-scan ultrasound and scanning laser ophthalmoscopy were performed to assess retinal.

Referenced severity assessment for PDR,the complexity score (CS) was calculated as the sum of five items:1. Extent of FVP: number of involved quadrants (1–4), awarding 1 point per quadrant;2. FVP location: involvement limited to anterior or limited to posterior to the equator scored 0; combined anterior + posterior involvement scored +1;3. TRD: +1 point when present;4. Traction-rhegmatogenous retinal detachment (TRRD): +2 points when present;5. Posterior vitreous detachment: absence of PVD contributed +1 point (17).

### 2.4 Surgical technique

All procedures were performed by the same experienced vitreoretinal surgeon using identical instruments throughout.A total of 96 patients participated and were randomly divided into two groups. Eyes in Group 1 (n=54) with significant cataracts initially underwent phacoemulsification of the crystalline lens plus the intraocular lens (IOL). A 25-gauge instrument was used to carry out anterior and central vitrectomy in all eyes. Additionally, an intravitreal injection of 0.1 mL of triamcinolone (Kenacort-A, DEVA,40 mg/mL) was administered to facilitate visualization and complete adherent posterior cortical vitreous detachment. All fibrovascular membranes were peeled to the greatest extent possible. The internal limiting membrane was not peeled in any of the eyes. After a thorough examination of the entire retina, retinal tears and lesion areas were treated with laser photocoagulation, followed by 360° panretinal laser photocoagulation. Total air-fluid exchange was performed, followed by the implantation of a dexamethasone implant into all eyes after the retina was fully reattached. Finally, HSO was injected into the eyes. Eyes in Group 2 (n=42) underwent the same procedure, except without intraoperative dexamethasone implantation (patients with severe fundus conditions underwent posterior capsulotomy and iridectomy). Silicone oil was removed from all the eyes after 3 months. In eyes with significant posterior lens capsule opacification, an intraoperative excision of the optical zone (approximately 5 mm in diameter) was performed. Postoperatively, the two groups of patients both received eyedrops postoperatively.

### 2.5 Subsequent visits

IOP was evaluated on the day after surgery, as well as at one week, one month, and three months postoperatively. Slit-lamp biomicroscopy was performed to evaluate the anterior chamber reaction. BCVA was evaluated at the first week, first month, and third month following the surgery, while CMT was measured using OCT at the same time point. B-scan ultrasonography and laser scanning fundus photography were performed to observe whether retinal redetachment occurred after surgery. Simultaneously, a slit-lamp biomicroscope was used to observe whether posterior iris synechia and posterior capsular opacification had occurred. Intraoperatively, the extent of the anterior PVR and other complications were observed under the wide-field retinal viewing system. Patients may have received anti-VEGF therapy if macular oedema developed during the observation period or received appropriate medication if the IOP was >21mmHg.

### 2.6 Outcome measures

To minimize the risk of assessment bias related to subjective outcome measures, all outcome assessors in this study were blinded. Assessors were not involved in patient group assignment and, blinded to allocation, conducted evaluations according to standardized procedures.The main outcome measures consisted of BCVA and IOP at the first, third month; anterior PVR; posterior iris synechia; the retinal reattachment rate at the third month; and the occurrence of anterior chamber fibrin exudation at the first week. The secondary outcome measures included changes in CMT, the number of anti-VEGF agents administered within 3 months postoperatively, and the degree of posterior capsule opacification (PCO). Adverse events such as AC haemorrhage, silicone microemulsion in the AC, and vitreous rehaemorrhage were also analysedanalyzed.

### 2.7 Statistical analyses

Data analysis was performed using SPSS 26.0 (SPSS Inc., Chicago, IL, USA). In this study, according to the types of data, the data are presented as the means, medians [IQR], standard deviations, and percentages. The analysis was conducted using the chi-square test, Shapiro-Wilk test, Fisher’s exact test, independent samples t-test, and nonparametric tests such as the Mann-Whitney U test or the Wilcoxon test. Kruskal–Wallis and Spearman. Statistical significance was defined as a *P* < 0.05. Difference Δ (Group 1 − Group 2) with 95% CIs is shown as the absolute difference in proportions (Newcombe method) for binary outcomes and as the Hodges–Lehmann median difference for continuous outcomes. *P* values are Bonferroni-adjusted (two-sided α = 0.05). We prespecified subgroup analyses by HbA1c (≤7.0%, 7.0–<9.0%, ≥9.0%) and age (≤51, 52–57, 58–63, >63 years). Three-month postoperative treatment effects on BCVA and anterior PVR were then estimated.

## 3 Results

### 3.1 Basic characteristics

**Figure 1:**
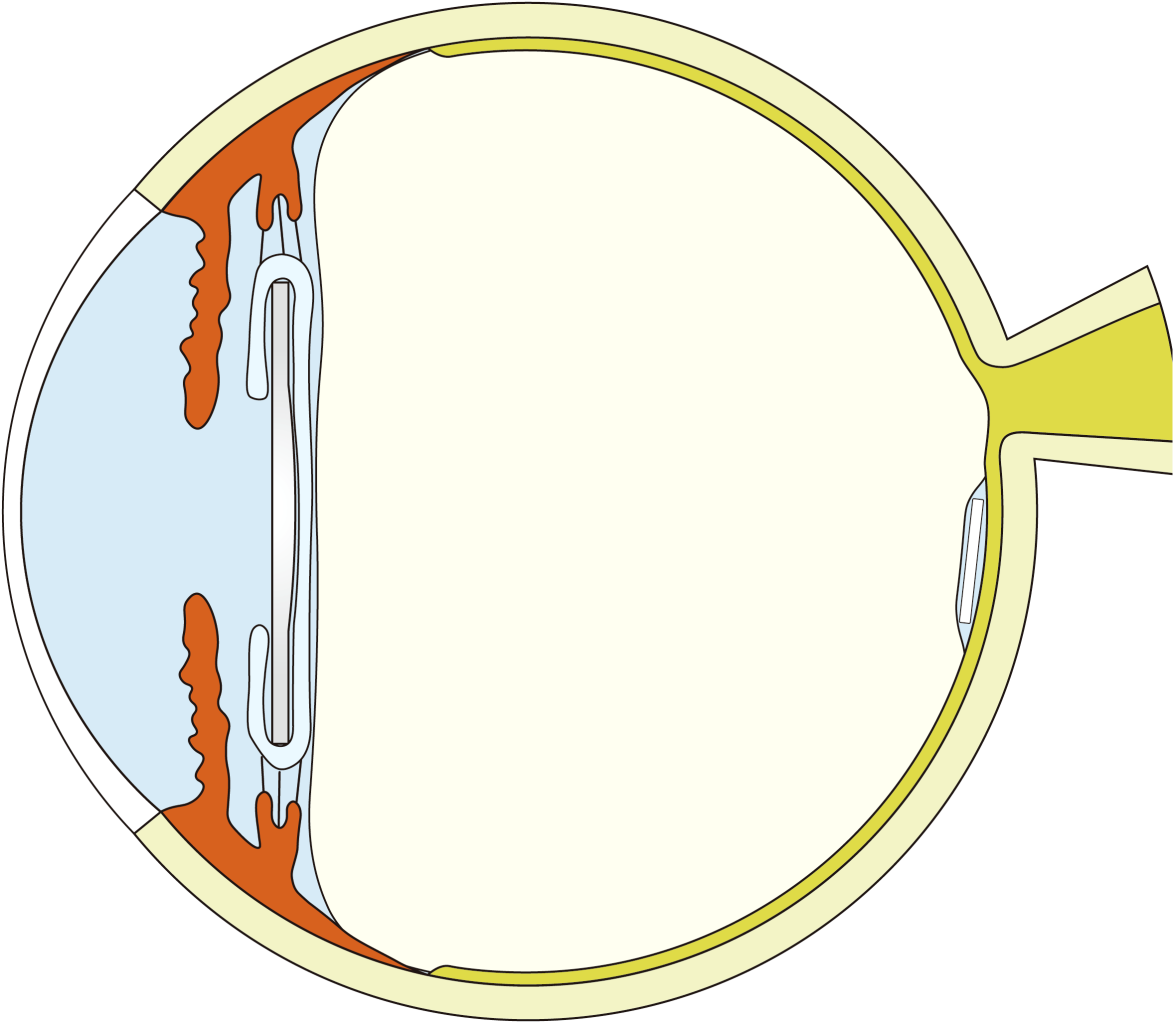
A schematic diagram: dexamethasone implant in a vitreous cavity filled with heavy silicone oil.

This prospective study comprised 96 eyes from 96 patients:54 eyes (54 patients) in Group 1 (PPV + HSO + dexamethasone implant,**Figure 1**) and 42 eyes (42 patients) in Group 2 (PPV + HSO). Ten patients were excluded from the final analysis because of failure to follow up on time, and six eyes were excluded because of dependence on silicone oil. The demographic and baseline characteristics are detailed in **Table 1**. No significant differences were observed between the two groups in age, sex, preoperative HbA1c levels, preoperative hypertension (*P* = 0.417, *P* = 0.823, *P* = 0.679, *P* = 0.340, respectively). The average preoperative BCVA was 1.70 [1.00, 1.70] in group 1 and 1.70 [1.06, 1.70] in group 2,*P* = 0.640.In group 1, the mean preoperative IOP was 16.75 ± 2.65 mmHg, while in group 2, it was 17.07 ± 2.54 mmHg, this difference was not significant (*P* = 0.583). Statistical analysis showed no statistically significant difference in the baseline CS scores between Group 1 and Group 2 (*P* = 0.898). Five patients in group 1 had IOLs, while three in group 2 had IOLs. The study included only patients who were above 40 years of age and had diabetes mellitus. The remaining 72 patients had cataracts and underwent phacoemulsification during vitrectomy. The surgical details between the two groups showed no significant difference (**Table 2**). The surgical duration was not significantly different between the two groups (*P* = 0.117). Due to the severity of their condition, 5 patients in Group 1 received perfluorocarbon liquid (PFCL) during surgery, 3 of whom underwent posterior capsulotomy and iridectomy. In Group 2, 3 patients also received PFCL during surgery, with 1 patient undergoing posterior capsulotomy and iridectomy.

**Table 1:** Patient demographic characteristics at baseline.

Characteristic	Group 1 (n=40)	Group 2 (n=40)	*P*
Age	57.78 ± 8.84	56.23 ± 8.15	0.417^d^
Gender, n (%)			0.823^c^
Male	19 (47.5)	18 (45)	
Female	21 (52.5)	22 (55)	
Preop HbA1c (%)	7.66 ± 2.08	7.85 ± 1.99	0.679^d^
Preop Blood pressure (mmHg)			
Systolic pressure	149.5[136.75,157.75]	146[134.0,161.0]	0.747^a^
Diastolic pressure	86 ± 9.24	84.1 ± 11.51	0.418^d^
Hypertension, n (%)	29 (72.5)	25 (62.5)	0.340^c^
Preop BCVA (logMAR)	1.7[1.00,1.7]	1.7[1.06,1.7]	0.640^a^
Preop IOP (mmHg)	16.75 ± 2.65	17.07 ± 2.54	0.583^d^
Postop HbA1c (%)	8.05 ± 1.97	7.85 ± 1.49	0.615^d^
The complexity score (point)			0.898^a^
2	3 (7.5)	6 (15)	
3	4 (10)	5 (12.5)	
4	13 (32.5)	8 (20)	
5	5 (12.5)	5 (12.5)	
6	12 (30)	8 (20)	
7	3 (7.5)	6 (15)	
8	0 (0)	2 (5)	
Lens status, n (%)			0.715^b^
Pseudophakic	5 (12.5)	3 (7.5)	
Phakic	35 (87.5)	37 (92.5)	
Aphakic eye	0 (0)	0 (0)	

Group-1: HSO filling during PPV surgery accompanied by dexamethasone implantation; Group-2: HSO filling during PPV surgery without dexamethasone implantation; ^a^Mann–Whitney U test; ^b^Fisher’s exact test; ^c^Pearson’s chi-square test; ^d^Independent samples t-test;BCVA, best corrected visual acuity; HbA1c, glycosylated haemoglobin; IOP, intraocular pressure; SD, standard deviation; n = 40 eyes per group.

**Table 2:** Surgical characteristics in group 1 and group 2.

Surgical Characteristic	Group 1 (n=40)	Group 2 (n=40)	*P*
Surgical duration (min)	53[48.25,60]	50[45,55]	0.117^a^
Use of PFCL, n (%)	5 (12.5)	3 (7.5)	0.715 ^b^
An iridectomy, n (%)	3 (7.5)	1 (2.5)	0.617^b^
Posterior capsulotomy, n (%)	3 (7.5)	1 (2.5)	0.617^b^
Phacoemulsification, n (%)	35 (87.5)	37 (92.5)	0.715^b^

Group 1: HSO filling during PPV surgery accompanied by dexamethasone implantation; Group 2: HSO filling during PPV surgery without dexamethasone implantation; ^a^Mann–Whitney U test; ^b^Fisher’s exact test; PFCL, Perfluorocarbon liquid; n = 40 eyes per group.

### 3.2 Visual acuity measurements

**Figure 2:**
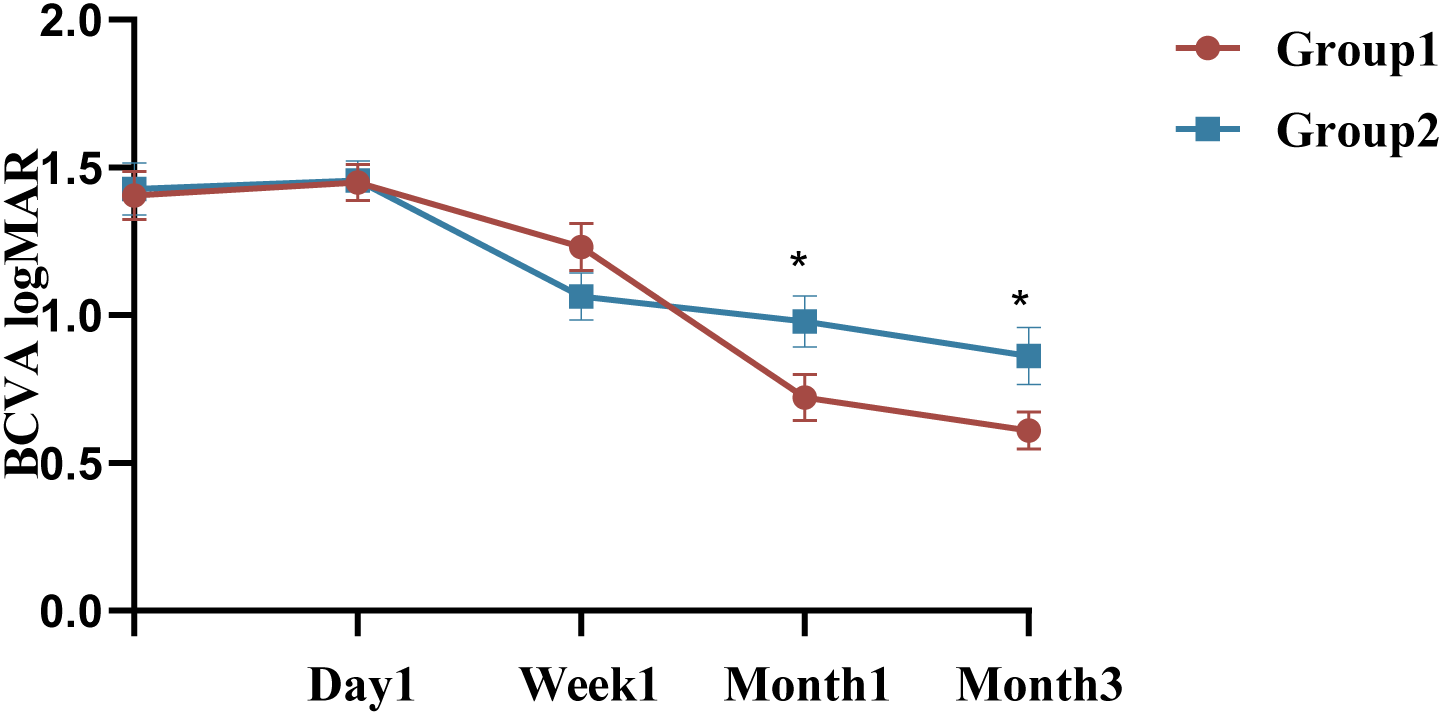
Changes and differences in BCVA between the two groups. BCVA: best corrected visual acuity; ^*^ *P* ≤ 0.05 (unadjusted); n = 40 eyes per group.

Both groups of patients showed improvement in vision postoperatively, with Group 1 experiencing a greater improvement in vision (**Table 3**, **Figure 2**). Group 1 had a mean preoperative LogMAR BCVA of 1.70 [1.00, 1.70], with significant improvements at the 1st week, 1st month, and 3rd month compared to baseline (all *P <*0.05,Bonferroni adjusted). Similarly, Group 2 had a mean preoperative BCVA of 1.70 [1.06, 1.70] LogMAR, with significant improvements in BCVA at the 1st week, 1st month, and 3rd month compared to baseline (*P <*0.001,Bonferroni adjusted). No significant difference in BCVA was observed between the two groups at baseline or 1 week postoperatively (*P* = 0.640, *P* = 0.135). However, at the first month, Group 1 showed a significantly greater improvement in BCVA than Group 2 (Group 1: 0.65 [0.30, 1.10], Group 2: 0.72 [0.60, 1.62], *P* = 0.040). At the third month, Group 1 demonstrated significantly BCVA compared to Group 2 (*P* = 0.046). However, after Bonferroni correction, neither the 1-month nor the 3-month postoperative comparisons were significant. In Group 1, 3-month BCVA differed across age strata (Kruskal–Wallis H = 9.902, *P* = 0.019), whereas treating age as a continuous variable showed no significant correlation with BCVA (Spearman ρ=−0.155, *P* = 0.339). In Group 2, 3-month postoperative BCVA differed across preoperative HbA1c strata (Kruskal–Wallis *P* = 0.039), whereas the overall correlation was not significant (Spearman ρ=−0.151, *P* = 0.354).

**Table 3:** BCVA in group 1 and group 2.

Time Point	Group 1 (n=40)	Group 2 (n=40)	Difference Δ (Group 1 -Group 2), 95% CI	*P^a^*	*P* (Bonferroni adjusted)
Preop BCVA (logMAR)	1.70[1.00,1.70]	1.70[1.06,1.70]	0.00 (-0.3, 0.18)	0.640	–
Postop BCVA(logMAR)					
1st week	1.16[0.85,1.70]	1.00[0.70,1.30]	0.18 (0.00, 0.39)	0.135	0.405
1st month	0.65[0.30,1.10]	0.72[0.60,1.62]	-0.22 (-0.45, 0.00)	0.040^*^	0.120
3rd month	0.40[0.30,0.82]	0.70[0.40,1.25]	-0.14 (-0.30, 0.00)	0.046^*^	0.138

Group-1: HSO filling during PPV surgery accompanied by dexamethasone implantation; Group-2: HSO filling during PPV surgery without dexamethasone implantation; ^a^Mann–Whitney U test BCVA: best corrected visual acuity; ^*^ *P* ≤0.05; n = 40 eyes per group; Confidence intervals are the conventional 95% CIs (unadjusted).

### 3.3 Intraocular pressure measurements

**Figure 3:**
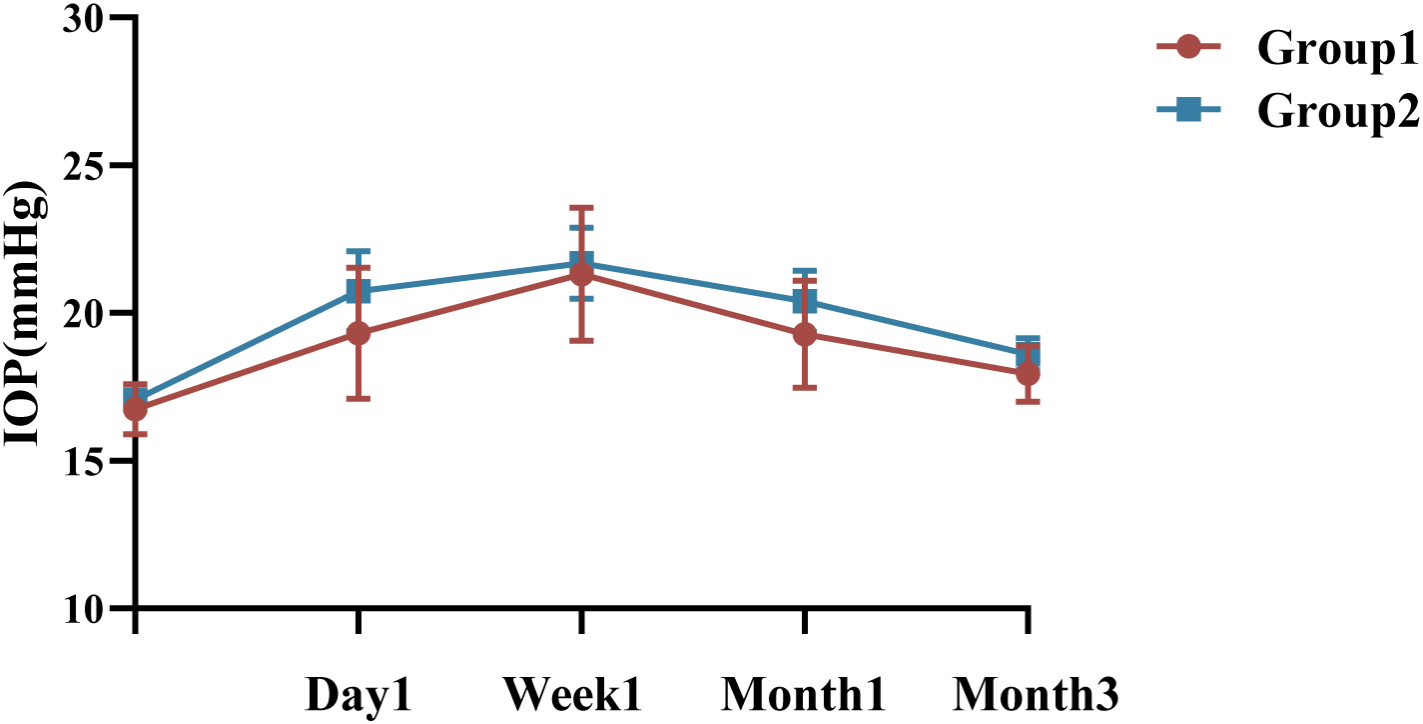
Changes and differences in IOP between the two groups. IOP, intraocular pressure; n = 40 eyes per group.

Group 1 had a mean preoperative IOP of 16.75 ± 2.65mmHg, with no significant variation in IOP at the 1st day, 1st month, or third month compared to baseline, *P >*0.05 (**Figure 3**). However, at the 1st week, the IOP in Group 1 was 19.75 [16.25, 24.40] mmHg, with a significant difference compared to baseline (*P <*0.05, Bonferroni adjusted). In contrast, the mean preoperative IOP in Group 2 was 17.07 ± 2.54 mmHg, with significant changes observed at all follow-up visits (*P <*0.05, Bonferroni adjusted). IOP measurements showed no statistically significant between Group 1 and 2 at any follow-up visit (*P* = 0.567, *P* = 0.721, *P* = 0.451, and *P* = 0.352, respectively), detailed summary is provided in **Table 4**. All patients with elevated IOP were treated with topical eye drops, and anterior chamber puncture was performed in a very small number of patients.

**Table 4:** IOP in group 1 and group 2.

Time Point	Group 1 (n=40)	Group 2 (n=40)	*P*
Preop IOP (mmHg)	16.75 ± 2.65	17.07 ± 2.54	0.583^d^
Postop IOP(mmHg)			
1st day	18.85[15.00,21.75]	19.70[13.68,25.78]	0.567^a^
1st week	19.75[16.25,24.40]	20.00[16.00,25.00]	0.721^a^
1st month	19.00[16.25,20.00]	19.50[16.00,21.00]	0.451^a^
3rd month	17.94 ± 2.95	18.61 ± 3.41	0.352^d^

Group-1: HSO filling during PPV surgery accompanied by dexamethasone implantation; Group-2: HSO filling during PPV surgery without dexamethasone implantation; ^a^Mann-W a:Mann–Whitney U tes; ^d^Independent samples t-test; n = 40 eyes per group.

### 3.4 Variables and safety related to treatment

**Figure 4:**
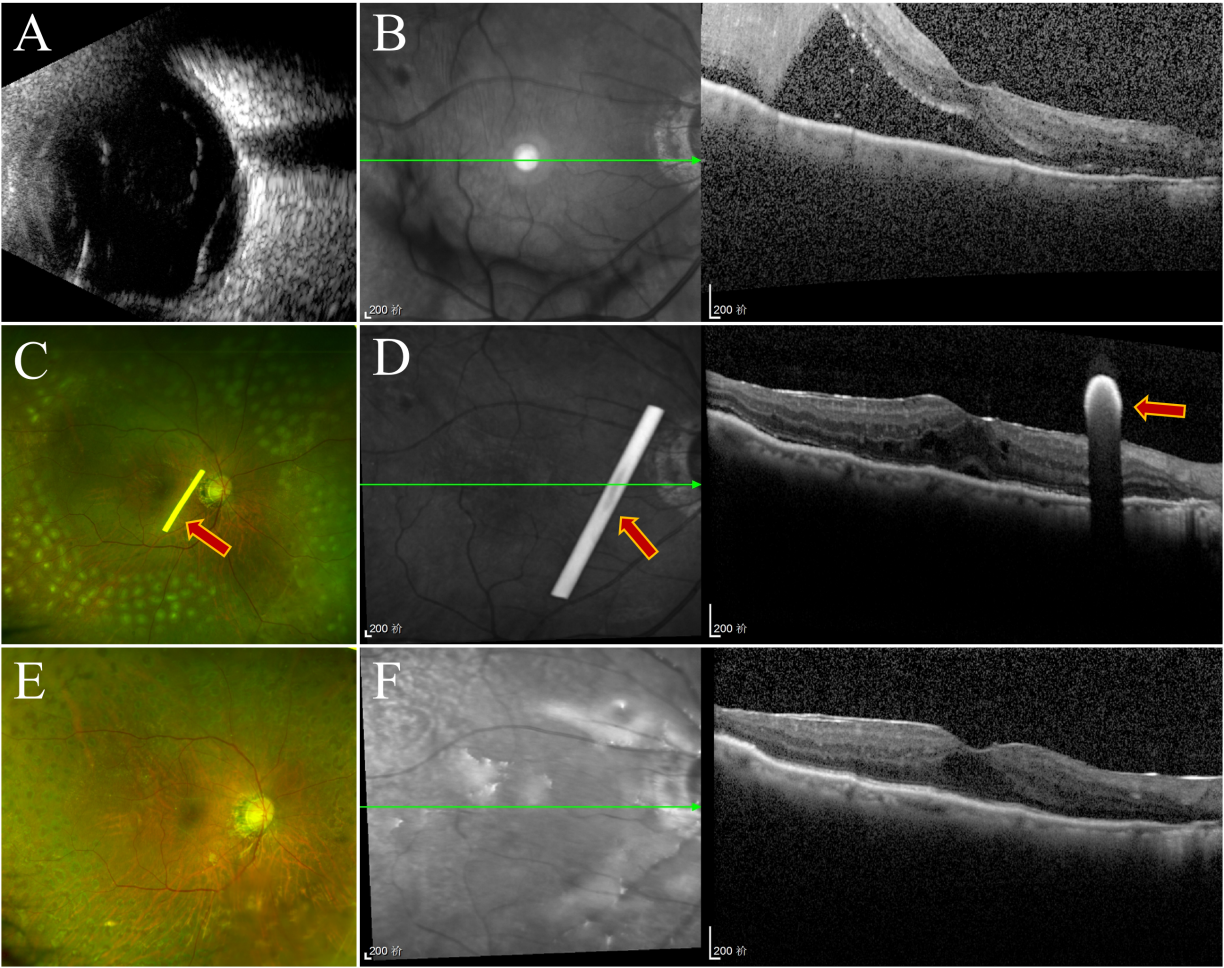
Follow-up examination of one patient in group 1. **(A)** Ultrasonography of the oculus dexter shows inferior retinal detachment. **(B)** Before surgery, OCT revealed retinal detachment. **(C)** SLO revealed silicone oil within the vitreous cavity, residual laser markings across the retina, and a white dexamethasone implant rod (marked with arrows). **(D)** OCT revealed that the retina was repositioned at the first week, and the dexamethasone implant was placed near the macular area (marked with arrows). **(E, F)** Three months later, the retina was repositioned, and the implant had been completely absorbed.

**Figure 5:**
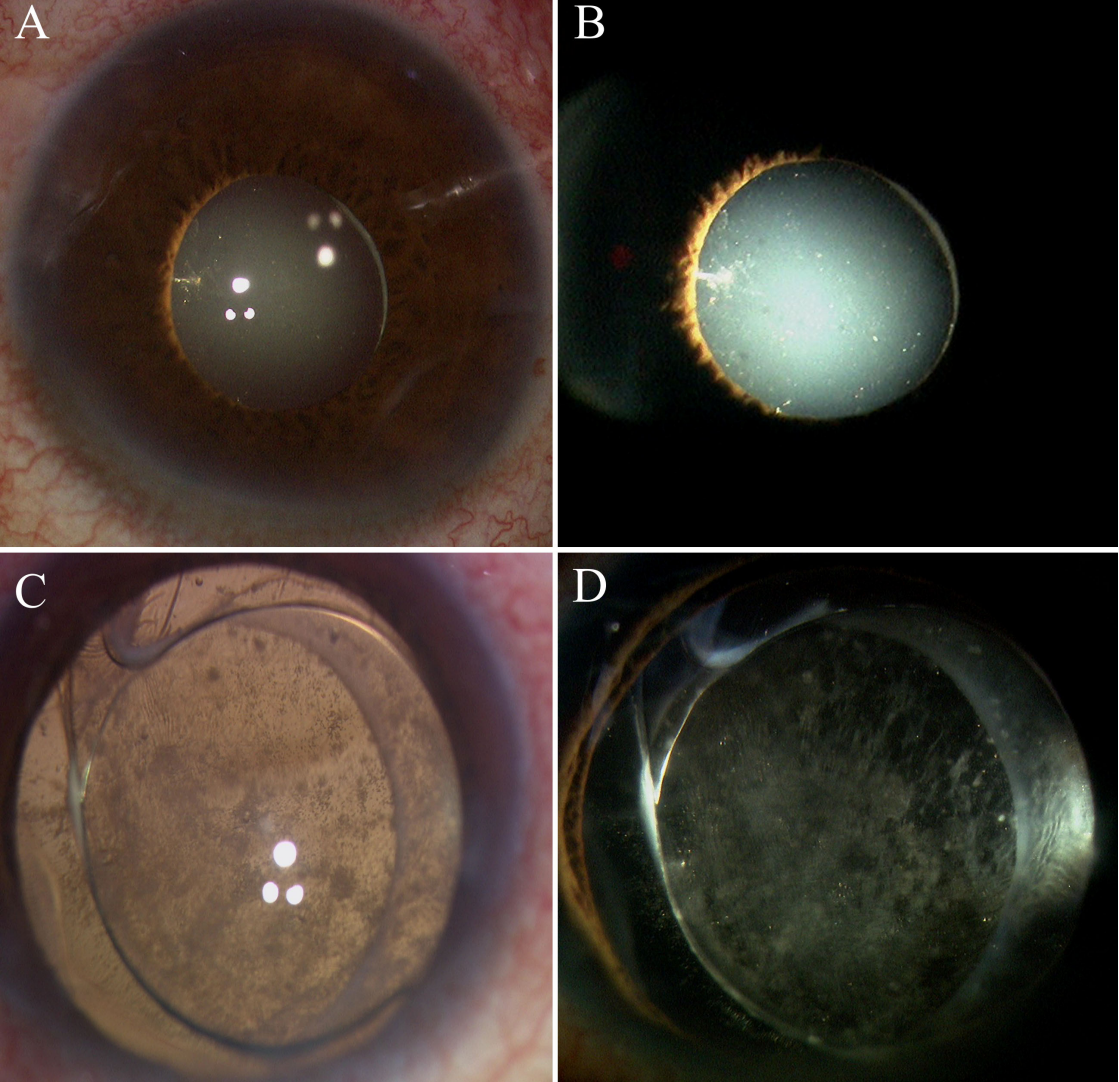
Intraoperative anterior segment observation in group 1 and group 2. **(A, B)** are from Group 1, and **(C, D)** are from Group 2. **(A, B)** No anterior proliferation was visible under the microscope during surgery. **(C, D)** Posterior capsule opacification and extensive anterior proliferative membranes were visible.

**Figure 6:**
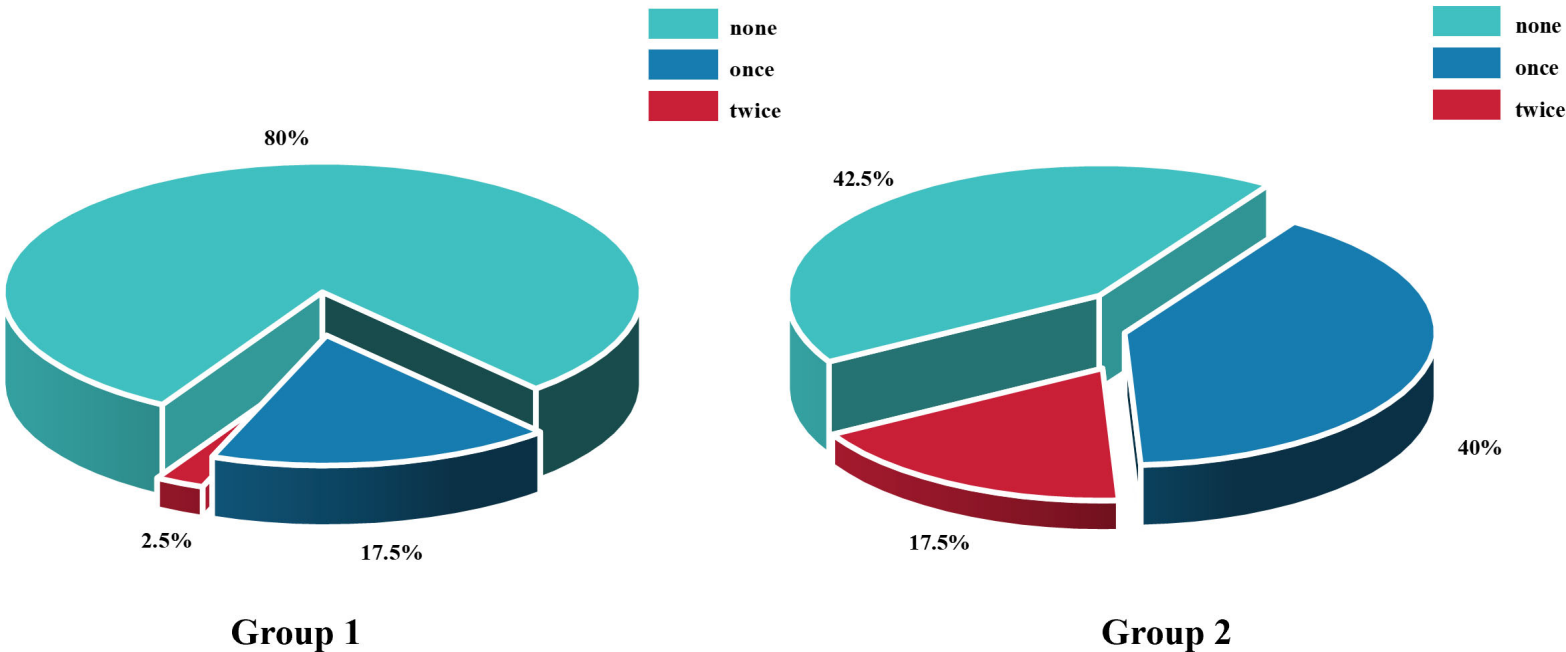
Comparison of the number of anti-vascular endothelial growth factor injections between Group 1 and Group 2 within 3 months postoperatively. n = 40 eyes per group.

The rate of retinal reattachment at the third month was 92.5% in Group 1 and 75% in Group 2 (*P* = 0.032)(**Figure 4**). After three months, the incidence of anterior PVR differed significantly between the two groups (*P* = 0.001), with a significant surgical effect in group 1 (**Figure 5**). Additionally, Group 1 presented significantly lower rates of anterior chamber fibrin exudation at the 1st week, as well as reduced posterior iris synechia at months 1 and 3 (*P* = 0.047, *P* =0.008). However, after Bonferroni correction, only anterior PVR remained statistically significant (*P* = 0.010). Additionally, in the postoperative IOP observations, no statistically significant difference was observed in the proportion of patients exhibiting IOP >21 mmHg between groups at the 1st day, 1st week, 1st month, or 3rd month (*P* = 0.228, *P* = 0.108, *P* = 0.576, *P* = 1.000, respectively). At 1 week postoperatively, patients in Group 1 who required 1, 2, or 3 types of glaucoma medications numbered 8 (20%), 3 (7.5%), and 2 (5%), respectively; in addition, 1 patient (2.5%) underwent AC paracentesis. In Group 2, the corresponding numbers were 4 (10%), 3 (7.5%), and 3 (7.5%), with no patients requiring AC paracentesis. Although the proportion of patients requiring glaucoma medications and invasive intervention was slightly higher in Group 1, the differences between groups were not statistically significant (*P* > 0.05). At 1 month postoperatively, intraocular pressure control showed a marked improvement compared to the 1-week postoperative period. In Group 1, 2 (5%), 3 (7.5%), and 1 (2.5%) patients required 1, 2, or 3 medications, respectively. In Group 2, the corresponding numbers were 2 (5%), 2 (5%), and 1 (2.5%). No patients in either group required AC paracentesis. There were no statistically significant differences between the two groups (*P* > 0.05). At 3 months postoperatively, only 2 patients (5%) in each group continued to require a single glaucoma medication, while all other patients achieved stable IOP without any treatment. At the third month, there was a significant difference in the number of anti-VEGF injections between the two groups.Especially among patients who received zero anti-VEGF injections, Group 1 had significantly more patients than Group 2, *P* = 0.003, after Bonferroni correction). (**Figure 6**). Other surgical complications did not differ significantly between the two groups, such as hyphema at the 1st month (*P* = 0.614), vitreous hemorrhage at the 1st month, 3rd month(*P* = 1.000,*P* = 1.000), and silicone microemulsion in the AC(*P* = 0.068). Detailed summary is provided in **Table 5**.

**Table 5:** Complications in group 1 and group 2.

Complication	Group 1 (n=40)	Group 2 (n=40)	Difference Δ (Group 1 -Group2), 95% CI	*P*	*P* (Bonferroni adjusted)
Retinal reattachment, n (%)	37 (92.5)	30 (75)	0.18 (0.02,0.33)	0.032^*b^	0.320^b^
Anterior PVR, n (%)	4 (10)	18 (45)	-0.35 (-0.53,-0.17)	0.001^*b^	0.010^*^ ^b^
Posterior capsule opacification, n (%)	10 (25)	20 (50)	-0.25 (-0.46, -0.05)	0.021^*c^	0.210^c^
Anterior chamber fibrin exudation, n (%)	1 (2.5)	9 (22.5)	-0.20(-0.34,-0.06)	0.007^**b^	0.070^b^
Posterior iris synechia, n (%)					
At first month	2 (5)	9 (22.5)	-0.18 (-0.32, -0.03)	0.047^*b^	0.470^b^
At third month	3 (7.5)	12 (30)	-0.23 (-0.39, -0.06)	0.008^**b^	0.080^b^
IOP elevation, (>21 mmHg), n (%)					
At first week	12 (30)	19 (47.5)	-0.18 (-0.39, 0.04)	0.108^c^	0.324^c^
At first month	7 (17.5)	9 (22.5)	-0.05 (-0.23, 0.13)	0.576^c^	1.000^c^
At third month	4 (10)	4 (10)	0.00 (-0.13, 0.13)	1.000^b^	1.000^b^
Number of anti-VEGF injections within 3 months, (%)					
none	32 (80)	17 (42.5)	0.38 (0.07, 0.61)	0.001^**^ ^b^	0.003**^b^
once	7 (17.5)	16 (40)	-0.23 (-0.47, 0.06)	0.047^*^ ^b^	0.141^b^
twice	1 (2.5)	7 (17.5)	-0.15 (-0.32, 0.04)	0.056^b^	0.170^b^
Hyphema,n(%)	3 (7.5)	1 (2.5)	0.05 (-0.05, 0.15)	0.614^b^	1.000^b^
Recurrent VH, n (%)					
At first month	1 (2.5)	2 (5.0)	-0.03 (-0.11, 0.06)	1.000^b^	1.000
At third month	2 (5.0)	3 (7.5)	-0.03 (-0.13, 0.08)	1.000^b^	1.000
Silicone microemulsion in the AC, n (%)	5 (12.5)	11 (27.5)	-0.15 (-0.32, 0.02)	0.068^c^	0.680

Group-1: HSO filling during PPV surgery accompanied by dexamethasone implantation; Group-2: HSO filling during PPV surgery without dexamethasone implantation; ^a^Mann–Whitney U test; ^b^Fisher’s exact test; ^c^Pearson’s chi-square test; ^d^Independent samples t-test; VH, vitreous hemorrhage; AC, anterior chamber; ^*^ *P ≤* 0.05;^**^ *P ≤* 0.01; n = 40 eyes per group; Confidence intervals are the conventional 95% CIs (unadjusted); P values were adjusted within outcome families using the Bonferroni method (FWER = 0.05): complications/anatomical outcomes m=10, IOP elevation m=3, and anti-VEGF injection counts m=3.

## 4 Discussion

To our knowledge, this is the first clinical trial investigating surgical HSO filling combined with intravitreal DEX implantation in PDR patients undergoing PPV. This study prospectively evaluated the effects of HSO tamponade in patients with diabetic TRD undergoing PPV and to evaluate the role of extended-release hormone implantation in controlling inflammation and mitigating postoperative complications. Our results suggested that the implantation of dexamethasone following intravitreal HSO filling in diabetic TRD patients undergoing PPV led to markedly reduce the incidence of anterior PVR and lower the number of anti-VEGF injections within 3 months postoperatively. It was also associated with improved postoperative visual acuity and higher retinal reattachment rates, as well as lower rates of AC fibrin exudation, posterior iris synechiae, and PCO.

Animal studies have demonstrated that oxygen and antioxidant gradients in the eye change following vitrectomy, affecting the circulation of inflammatory cells (18). Furthermore, complex ocular surgery and increased risk factors for inflammation can result in increased levels of inflammatory factors (19, 20). Moreover, blood glucose level also plays an important role in the development of DR. In diabetic patients, elevated blood glucose can disorder the blood-aqueous barrier after surgery, leading to increased vascular permeability, elevated inflammatory mediators (21). But we only observed that in Group 2, 3-month postoperative BCVA differed across preoperative HbA1c, whereas the overall correlation was not significant. We think the short duration of follow-up and insufficient sample size limited our observations. Silicone oil is increasingly commonly utilized in patients with diabetes, RD, and PVR. Silicone oil can help PDR patients prevent recurrent haemorrhage, reset detached retinas, and suppress the development of PVR (22–24). Additionally, during the surgery, the operator will select light silicone oil (LSO) or HSO filling on the basis of the location of the retinal rupture and detached surface, the existence of PVR, and the patient’s overall health (25). Although silicone oils can help with disease recovery, they also increase the levels of vascular permeability-related inflammatory factors, which promote the development of ME (26). HSO is more prone to emulsification and irritation than regular silicone oil (27). Some investigations reported mild to moderate AC reactions, including fibrous membranes, keratic precipitates, pseudohypopyon, and hyphaema, with HSO filling for three months (28), Li W et al. observed that HSO can induce posterior chamber inflammatory reactions, such as the production of cataracts and deposits on the lens surface (29, 30). Morescalchi F proposed that HSO induces an inflammatory response by increasing cytokine levels in the vitreous cavity and that the cytokines involved are similar to those involved in PVR (31). The primary cause of recurrent RD in patients with diabetic TRD combined with cataracts is PVR and high levels of inflammatory factors. M. Sherif et al. retrospectively evaluated five cases of recurrent RD with stage C PVR and concluded that dexamethasone could be a therapeutic option for PVR (32). In this study, it was observed that 45% of patients who underwent HSO tamponade had anterior PVR at 3rd month. Utku Limoni et al. reported that implanting dexamethasone combined phacoemulsification and PPV in patients with diabetic TRD had a stable effect, confirming the importance of long-acting steroid dexamethasone implants (16). This research also confirmed that compared to Group 2, patients who received dexamethasone implantation had better postoperative outcomes in controlling anterior segment inflammation, reducing the probability of PVR and RD. PVR most commonly occurs within one month after RD surgery (77%) and by approximately 45 days in 95% of cases—indicating a relatively acute process (33). And anterior PVR can be identified and evaluated more effectively at the time of silicone oil removal. However, as some PVR and RD may arise over extended follow-up, our study has inherent limitations in evaluating long-term outcomes. Compared to LSO, HSO exhibits a greater tendency to emulsify due to its lower viscosity (27). This leads to a significantly increased accumulation of oil globules in the AC, on the iris surface, and along the posterior surface of the cornea. Similarly, in our study, numerous silicone oil globules were observed on the posterior surface of the lens capsule in many patients during HSO removal surgery. These globules not only exacerbated intraocular inflammation but also contributed to the development of anterior PVR and PCO, as well as elevated IOP. The incidence of HSO microemulsions in the AC was 12.5% in patients who received dexamethasone implants, compared to 27.5% in those without the implant. This difference is presumed to be associated with the anti-inflammatory properties of dexamethasone. In addition, no detailed assessment was conducted in this study, and further improvements are needed moving forward.

Clinical studies have shown that the use of Ozurdex^®^ after vitrectomy is safe and effective in reducing DME (34–36). Hsu and Cherng-Ru reviewed 12 eyes and confirmed that the DEX implant could help manage recurrent macular oedema (ME) in patients who had undergone vitrectomy with silicone oil filling (37). Additionally, Altun reported that administering a dexamethasone implant and silicone oil filling during vitrectomy in individuals with proliferative PDR not only reduced recurrent DME but also decreased the number of anti-VEGF agents (38). The majority of patients in this study had VH and TRD preoperatively; thus, we were unable to collect preoperative CMT values. However, by comparing the number of anti-VEGF injections administered three months postoperatively, we found a significant difference between the two groups (*P <*0.001). Additionally, we observed that the CMTs of some patients in Group 1 remained lower than their values from the first week.The per-treatment cost of the dexamethasone implant is comparable to that of other anti-VEGF agents. However, due to its sustained-release properties, the implant may reduce the need for frequent injections, thereby decreasing overall treatment burden and associated costs. Furthermore, it provides a valuable option for patients with low follow-up adherence.

Dexamethasone implants are known to cause an increase in IOP and accelerate cataract progression. Therefore, all patients included in this study underwent phacoemulsification with vitrectomy, with postoperative IOP monitoring. The results revealed a statistically significant difference in IOP at the first week between Group 1 and the baseline level (*P <*0.001). Compared with the baseline IOP, Group 2 showed a marked statistical difference at different time points (*P* < 0.05). The IOP reached its peak at the first week in both groups, which may be attributed to the higher rate of retinectomy. The disruption of the blood–ocular barrier during surgery likely led to an increase in inflammatory factors, which are consistent with those reported by Wong D et al. (39). However, IOP did not differ significantly between two groups at the first day, first week, or months 1 or 3. Patients with IOP >21 mmHg were counted separately, and no significant differences were observed between the two groups at any of the time points. The majority of patients in this study with elevated IOP were treated with topical eye drops, and their IOP was effectively controlled. Moreover, our results show that, over time, the number of patients requiring pharmacologic therapy and AC paracentesis gradually decreased. The 3-month postoperative follow-up indicates that the dexamethasone implant appears to be safe.This result is consistent with the findings of the Ozurdex PLACID study group and a 12-month multicentre prospective clinical trial, which demonstrated that dexamethasone implants cause transient IOP increases that can be managed with medication (40, 41). Silicone oil is not only toxic to the retina but also increases the risk of postoperative complications with prolonged filling time (42). Before surgery, the doctor examines the peripheral retina and vitreous. For patients with a favourable recovery, silicone oil is generally removed after three months. The development of PCO is influenced by multiple factors, including blood glucose levels, inflammatory mediators, IOL types, and the effect of silicone oil. Considering that anterior PVR may interfere with the assessment of the posterior capsule, we believe that although a difference in the incidence of PCO was observed between the two groups in this study, it cannot be conclusively attributed to the anti-inflammatory effects of dexamethasone.

Based on the above discussion, the occurrence of complications such as anterior PVR, PCO, and SO emulsification is influenced by many complex factors (21, 43). Compared with HSO, LSO tamponade may be associated with a lower incidence of anterior PVR and silicone oil emulsification.Considering the factors outlined above,we plan to include a LSO control group in future studies to systematically investigate the effects of dexamethasone implants on key outcomes.This will allow for a more in-depth exploration of whether dexamethasone can effectively suppress the inflammatory responses potentially triggered by HSO itself. Furthermore, in view of the potential for late postoperative PVR and recurrent RD, we have planned a multicenter, prospective clinical trial with 12-month follow-up that includes a LSO control group.

## 5 Conclusion

To the best of our knowledge, this is the first study to evaluate the effect of dexamethasone implants in eyes with HSO tamponade in patients with PDR. Our preliminary findings suggest that intraoperative use of dexamethasone implants may be a safe and potentially beneficial adjunctive treatment strategy for diabetic TRD patients undergoing phacoemulsification combined with vitrectomy and HSO tamponade. Over the 3-month postoperative observation period, the dexamethasone implant demonstrated potential in reducing inflammatory responses and decreasing the need for postoperative anti-VEGF medications. The findings of this study provide a valuable direction for future research.

## Data Availability

The original contributions presented in the study are included in the article/supplementary material. Further inquiries can be directed to the corresponding authors.
